# Interactions of arbuscular mycorrhizal and endophytic fungi improve seedling survival and growth in post-mining waste

**DOI:** 10.1007/s00572-017-0768-x

**Published:** 2017-03-20

**Authors:** Katarzyna Wężowicz, Piotr Rozpądek, Katarzyna Turnau

**Affiliations:** 1 Institute of Environmental Sciences, Jagiellonian University, Gronostajowa 7, 30-387 Kraków, Poland; 2 Malopolska Centre of Biotechnology, Jagiellonian University, Gronostajowa 7A, 30-387 Kraków, Poland

**Keywords:** AMF, Fungal endophytes, *Verbascum lychnitis*, Toxic metals, Photosynthesis efficiency

## Abstract

The impact of fungal endophytes and the modulating role of arbuscular mycorrhizal fungi (AMF) on the vitality of *Verbascum lychnitis*, grown in the laboratory in a substratum from a post-mining waste dump was investigated. We report that inoculation with a single endophyte negatively affected the survival rate and biomass production of most of the plant-endophyte consortia examined. The introduction of arbuscular mycorrhiza fungi into this setup (dual inoculation) had a beneficial effect on both biomass yield and survivability. *V. lychnitis* co-inoculated with AMF and *Cochliobolus sativus*, *Diaporthe* sp., and *Phoma exigua* var. *exigua* yielded the highest biomass, exceeding the growth rate of both non-inoculated and AMF plants. AMF significantly improved the photosynthesis rates of the plant-endophyte consortia, which were negatively affected by inoculation with single endophytes. The abundance of PsbC, a photosystem II core protein previously shown to be upregulated in plants colonized by *Epichloe typhina*, exhibited a significant increase when the negative effect of the fungal endophyte was attenuated by AMF.

**Electronic supplementary material:**

The online version of this article (doi:10.1007/s00572-017-0768-x) contains supplementary material, which is available to authorized users.

## Introduction

Plants, in their natural environments, are a refuge for numerous organisms ranging from small mammals, through invertebrates and fungi, to unicellular bacteria (Beckett et al. 1992; Lindow and Brandl 2003). From the functional standpoint, a single plant can be referred to as a specific “microcosmos,” where structure and functions are organized by a dense net of multidimensional interactions between the plant and its inhabitants, as well as by interactions between the organisms colonizing the plant (Van der Putten et al. 2001; Singh et al. 2004; Felton and Tumlinson 2008; Aly et al. 2011). The symbiosis of plants has been the subject of a multitude of studies. Microorganisms, fungi, and bacteria take central positions in this field of symbiosis due to their impacts on the host’s biology and potential application.

Microorganisms inhabiting the plant differ in their trophic strategy ranging from pathogens and parasites to mutualists (Redman et al. 2001). Additionally, the character of the relationship of a specific microorganism and its host plant is variable and may change over time. In many cases, abiotic and biotic constraints such as resource availability, herbivores, factors limiting carbon assimilation, and stage of ontogeny among others may elicit a switch from mutualism to parasitism and vice-versa (Kiers et al. 2010). In plant symbiotic interactions, the microorganism functions as an additional sink for carbohyrates. In instances where the costs of maintaining a fungal partner do not exceed benefits, the plant acts as the most effective provider to the fungus. However, under environmental challenges, a trade-off in resource allocation may limit carbon flow from the plant to its symbiotic partner and disturb the mutualistic equilibrum (Ahlholm et al. 2002; Bever et al. 2009). This phenomenon, described for symbiotic fungi, is referred to as the endophyte-parasite continuum (Schulz and Boyle 2005; Mandyam et al. 2013; Reininger and Schlegel 2016). Additionally, the presence of fungi and bacteria with pest control capabilities may influence the lifestyle of co-inhabitants. This ability of symbiotic microorganisms may be exploited as an environmentally friendly alternative for traditional pest control in agriculture (Doan et al. 2008; Jäschke et al. 2010).

The growing deposition of toxic metals in the environment severely restricts plant growth. This has serious economic and social implications, due to yield losses in agriculture, contamination of water reservoirs, and increased incidence of diseases. A promising method for restoring areas polluted by industrial activities is phytoremediation. Phytoremediation utilizes the ability of plants to remove and/or immobilize a wide range of organic and inorganic pollutants, including toxic metals deposited in the substratum (Turnau et al. 2006).

The remedial capacity of plants can be greatly improved by microorganisms such as bacteria and fungi. There are two major classes of ubiquitous fungal symbionts associated with plants in terrestrial ecosystems: arbuscular mycorrhizal fungi (AMF) and fungal endophytes. The roots of more than 80% of plants on land are colonized by AMF (Smith and Read 2008). The benefits related to mycorrhization include increased root absorption area and hence increased uptake of nutrients (Smith and Read 1997a, 1997b), a better tolerance to water stress (Porcel and Ruiz-Lozano 2004), high salinity (Daei et al. 2009), pathogens (Cordier et al. 1998), and toxic metals (Turnau et al. 2006). Endophytes are commonly isolated from roots and shoots of plants (Stone et al. 2000; Zhang et al. 2006; Wężowicz et al. 2014). Their presence within host tissues may promote plant growth, nutrient acquisition, and confer tolerance against abiotic and biotic stresses (Redman et al. 2002; Lewis 2004; Schulz and Boyle 2005; Waller et al. 2005; Hiruma et al. 2016), including toxic metal tolerance (Deng et al. 2011; Li et al. 2011a; Li et al. 2011b; Li et al. 2012). Fungal endophytes have been shown to possess a remarkable ability to accumulate toxic metals: the DSE (dark septate endophyte) *Exophiala pisciphila* (H93) from the roots of *Urundinella bengalensis* accumulated over 5% Cd in its dry mass (Zhang et al. 2008). Interestingly, DSEs are ubiquitous and colonize roots of plants growing in extremely unfavorable, post-industrial environments. Furthermore, their colonization increases with the growing quantity of toxic metals in the substratum (Regvar et al. 2010; Shen et al. 2015; Zhao et al. 2015).

Excess of toxic metals alters photosynthesis functions at various levels of organization. For instance, disturbances in chlorophyll biosynthesis and thylakoid electron transport are induced in the presence of Pb, Zn, and Cd (Tripathy and Mohanty 1980; Sheoran et al. 1990; Stefanov et al. 1995; Vodnik et al. 1999; Villiers et al. 2011). As a result, plant growth and development is severely impaired. Symbiosis with fungi was reported to attenuate the effects of toxic metals stress. Inoculation with fungal endophytes increased photosynthetic pigment abundance and enhanced water use efficiency, promoting photosynthesis and rice growth in Pb stress conditions (Li et al. 2012). Mycorrhizal rice showed higher chlorophyll content and photosynthetic efficiency in the presence of As, in comparison to non-mycorrhizal plants (de Andrade Lopez et al. 2015). AMF were shown to upregulate detoxification mechanisms including the antioxidant system. Efficient ROS (reactive oxygen species) scavenging is crucial to maintaining proper function of electron transport and subsequent ATP and NADPH production in the photosynthetic electron transport chain. Improved photosynthetic efficiency was attributed not only to the protective role of the fungi, but also, according to Rozpądek et al. (2015), to symbiont-related alterations in the composition, i.e. the abundance of specific photosystem proteins, including PsbC and Lhcb3 that may positively impact the function of the photosynthesis apparatus.

So far, AMF-plant or endophyte-plant interactions were studied in single plant-fungus models. The few co-inoculation studies, where plants were inoculated by more than one group of fungi, were performed using grasses as the host plants. The results of these experiments are not ambiguous, showing that the microorganisms inhabiting plant hosts positively influenced plant growth (Mack and Rudgers 2008; Scervino et al. 2009; Larimer et al. 2012).

AMF have been described as important factors in remediation strategies (Leyval et al. 1997; Turnau et al. 2005). The role of endophytes in this process also has been well demonstrated (Weyens et al. 2009 and publications cited there; Soleimani et al. 2010; Li et al. 2012 and publications cited there). Nevertheless, the majority of research related to the use of endophytes in phytoremediation comes from studies of bacterial endophytes. Endophytic fungi are yet to receive the appropriate attention from the scientific community. In a previously published article, the diversity of fungal endophytes from *Verbascum lychnitis*, a biennial herb from the Scrophulariaceae family, inhabiting post-industrial wastelands in South Poland was shown (Wężowicz et al. 2014). The aim of this study was to evaluate the growth response, photosynthesis performance, and possible alterations in the composition of selected photosynthetically active pigments and structural proteins of *V. lychnitis* inoculated with selected endophytic fungi and co-inoculated with endophytes and AMF. The plants were cultivated in the substratum of the mine dump “Chrzanów” under laboratory conditions. This permitted selection of fungal endophytes with the highest potential for utilization in phytoremediation. We predicted that the isolated fungal endophytes would affect their host differently and their interaction would be modulated by AMF.

## Materials and methods

### *V. lychnitis* seed germination

Seeds used in the experiment were collected in autumn 2010 from *V. lychnitis* plants growing on the post-mining waste dump of the Trzebionka Mining Company (ZG Trzebionka) in close proximity to Chrzanów, PL (50^o^ 09’ 19″ N, 19^o^ 25’ 10″ E). Seeds were husked from capsules under a binocular microscope and surface sterilized with 8% NaOCl (5 min), 96% (1 min) and 75% EtOH (3 min; Achary et al. 2012, modified). Subsequently, seeds were rinsed for 1 min 5 times in deionized water, dried on sterile filter paper, and germinated on water agar in Petri plates at room temperature, 12 h photoperiod and light intensity ca. 120 μmol × m^−2^ × s^−1^ of PAR (photosynthetically active radiation).

### Inoculation with endophytes

Ten-day-old seedlings were transferred to Petri plates (10 per each plate) filled with modified Strullu-Romand (MSR) medium (the concentration of GelGro™ (MP Biomedicals, FR) was increased to 11.0 g × L^−1^ instead of 3.0 g/L) without sucrose. Small plugs (2 × 2 mm) of fresh endophyte mycelium were placed under the roots of seedlings. Plants were inoculated with one of the following endophytes: *Pyrenophora leucospermi* Crous and L. Swart, *Cadophora* sp., *Diaporthe* sp., *Cochliobolus sativus* (E.J. Butler) Shoemaker, *Myrothecium* sp., *Xylaria* sp., *Phialophora mustea* Neerg*.*, and *Phoma exigua* var. *exigua* Desm. All fungi were isolated from shoots or roots of *V. lychnitis* plants (Wężowicz et al. 2014) and had been stored at 4 °C in the Małopolska Center of Biotechnolgy. Seedlings were grown with the endophytes for 28 days in a growth chamber (Percival AR-41L2X) at 21 °C, 12 h photoperiod, 60% humidity, and light intensity ca. 120 μmol × m^−2^ × s^−1^ of PAR.

### Substrate preparation

The substrate was collected from a deep layer (1 m depth) of the mine tailings, from sites devoid of plant cover for several years. The substratum was stored for 2 years before the experiment. The substratum contained practically no organic matter, was P- and N-deficient (*P*_total_ − 900 μg × g^−1^, *P*_available_ 114 μg × g^−1^ N − 900 μg × g^−1^) and contained elevated quantities of toxic metals. The total Zn concentration reached 12,000 μg × g^−1^ (ca. 3.8 μg g^−1^ after extraction with Ca(NO_3_)_2_, Pb − 2400 μg × g^−1^ (1.9 μg × g^−1^ after extraction with Ca(NO_3_)_2_), and Cd − 100 μg × g^−1^ (1.4 μg × g^−1^ after extraction with Ca(NO_3_)_2_ (Orłowska et al. 2005). The pH was slightly alkaline (pH 7.4). The substrate was not sterilized in order to replicate conditions in the tailings and also to investigate the possibility of use of the inoculum in recultivating the mine dump. The growth medium comprised the substrate, sterile sand, and expanded clay in a 3:1:1 (*v/v/v*) ratio and was supplemented with rock phosphate (1 g × L^−1^).

### AM fungal inoculum preparation

*Rhizopagus irregularis* (UNIJAG.PL.32 sub-cultured from BIORIZE E-1-99/Lav) inoculum, containing ca. 20 spores per gram of growth medium, was produced in pot cultures according to Jurkiewicz et al. (2010).

### Experimental design

Seedlings were transferred to pots (182 cm^3^) filled with prepared growth medium (as described above). Where expected, AM fungal inoculum (2 g per pot) was added prior to seedlings transfer. The following treatments were prepared: (1) control seedlings—without mycorrhizal inoculum or endophytes (25 pots; NM controls); (2) seedlings inoculated with a single endophyte species (25 pots per each endophyte); (3) seedlings inoculated with an endophyte and *R. irregularis* (25 pots per each endophyte); and (4) seedlings inoculated with *R. irregularis* alone (25 pots). In each pot, a single plant was placed. Pots were irrigated once with 15 ml KNO_3_ solution (1.212 g/L) and cultivated in sealed sunbags (Sigma-Aldrich) in a greenhouse at 20 °C, illuminated for 12 h with 120 μmol × m^−2^ × s^−1^ of PAR. The pots were placed in trays, and the trays were rotated twice a week (each tray was rotated clockwise). The cultures were irrigated once per 2 weeks with 15 ml of Long Ashton solution (Seddas-Dozolme et al. 2010) without phosphorus.

### Biometric measurements

After 24 weeks of cultivation, plants were removed from pots and weighted. Roots were carefully washed in tap water before weighing. Survivability was calculated as frequency of plants (out of 25) that survived at the end of the experiment. Survivability data was arranged in a contingency table, and the chi-square test was used to evaluate statistical significance versus the NM controls. Roots were stained to visualize and assess mycorrhizal and endophyte colonization. Survivability and biomass production results were used to select plant-fungi consortia for analysis of photosynthesis efficiency, pigment/protein abundance, and the impact of the presence of endophytic fungi on the development of mycorrhiza.

### Measurement of chlorophyll *a* fluorescence transient OJIP

Prior to plant harvesting, chlorophyll *a* fluorescence transients OJIP were measured with a Handy PEA-fluorimeter (Hansatech Instruments, UK). Chlorophyll fluorescence is widely used to assess plant vitality (Strasser et al. 2004). The transients were induced by red light (peak at 650 nm) of 3.000 μmol photons m^−2^ × s^−1^ provided by an array of three light-emitting diodes and recorded for 1 s with 12 bit resolution. Data acquisition was performed every 10 μs (in the interval from 10 μs to 0.3 ms), every 0.1 ms (0.3–3 ms), every 1 ms (3–30 ms), every 10 ms (30–300 ms), and every 100 ms (300 ms to 1 s). The measurements were conducted on intact plants (8–15 replicates per treatment), dark-adapted for a minimum of 25 min prior to measurement.

For each treatment, the average OJIP fluorescence transient was analyzed according to the JIP test (Strasser et al. 2004), with the BIOLYZER5PHP software (http://www.fluoromatics.com). The parameters chosen to be calculated in the present study were the performance index (PI_ABS_), specific energy fluxes calculated per reaction center (TRo/RC, ETo/RC, ABS/RC, RC/AB, DIo/RC**)**, area, quantum efficiencies, and flux ratios (ϕ_Eo_, ϕ_Po_, Ѱo). For parameter descriptions, see Table 1.

**Table 1:** Terms used in the analysis of the OJIP fluorescence transient

Symbol	Description
PI_ABS_	The performance of the photosynthesis apparatus expressed in relation to absorption. Accounts for the density of reaction centers, the quantum yield of primary photochemistry, and the ability to transfer electrons from PSII to PSI
TR_o_/RC	Energy capture by one reactive center
ET_o_/RC	Rate of the energy transfer by one reactive center
ABS/RC	The effective antenna size expressed as absorbance per reaction center
DI_o_/RC	The amount of energy dissipated per active reaction center
Area	It is proportional to the concentration of electron acceptor pool size of PSII that includes QA, QB, and PQ
QA	Primary quinone-type electron acceptors of PSII
QB	Secondary quinone-type electron acceptors of PSII
PQ	Plastoquinone-a quinone electron transporter
ϕ_Eo_ (ETo/ABS)	The probability that an absorbed photon will move an electron into the electron transport chain
ϕ_Po_ (TRo/ABS)	The maximal yield of primary photochemistry. When calculated from extreme values (Fo and Fm) Fv/Fm
Fo	Fluorescence emitted when all reaction centers are open
Fm	Fluorescence emitted when all reaction centers are closed
Fv/Fm	Current photochemical capacity of PSII
Ѱ_0_ (ETo/TRo)	Quantum yield of electron transport

### AM fungi and endophyte colonization assessment

Six plants and 15 fragments of each root system per treatment were evaluated for fungi colonization. The roots were stained according to the modified Phillips and Hayman (1970) method. After being washed in running tap water, roots were incubated in 10% KOH solution for 24 h and rinsed in water, acidified in 5% lactic acid solution for 1 h, and stained with 0.05% aniline blue in 80% lactic acid for 24 h at room temperature. Finally, the material was transferred to 80% lactic acid for storage. For colonization assessment, roots were cut into 1.5 cm fragments and mounted on slides in glycerol/lactic acid (5:1). Analyses were carried out using a Nikon Eclipse 800 (Japan) microscope. Mycorrhizal colonization assessment was carried out according to Trouvelot’s method (Trouvelot et al. 1986, http://www.dijon.inra.fr/bbceipm/Mychintec/Mycocalc-prg/download.html). Observations of endophyte presence in roots were carried out simultaneously with mycorrhizal colonization assessment. Where necessary, to visualize the endophyte, additional staining with Sudan IV (3 g in 740 ml 95% ETOH and 240 ml dH_2_O) was carried out on roots previously stained as above.

### SDS-PAGE and immuno-blotting

To verify if fungi induced photosynthesis efficiency changes dependent on reorganization of photosyntem II antennae protein composition, the abundance of Lhcb3 which is one of three major chlorophyll a/b binding proteins in photosystem II and the antennae Psbc protein was assessed. Additionally, the abundance of Rubisco LSU (large subunit), which catalizes the incorporation of CO_2_ into the Calvin-Asada cycle, was measured. Sample preparation was performed according to Rozpądek et al. (2015) with modifications. Protein separation was performed in a 4% stacking gel (pH 6.8) and 12% resolving gel (pH 8.8) with 10 μg of PsbC or Rubisco LSU or 20 μg of Lhcb3 protein loaded per lane. Electrophoresis was carried out at constant 24 mA for the first 15 min (in the stacking gel), followed by 36 mA until full separation of the protein marker ladder (Thermoscientific, LT). Following electrophoresis, transfer to PVDF membranes (semi-dry) was performed using Trans-Blot Turbo Transfer System (BioRad, US). For immunodetection, Agrisera (SE) primary polyclonal antibodies in the following dilutions were used: PsbC (AS11 1787) 1:2500, Rubisco LSU (AS03 037) 1:5000, and Lhcb3 (AS01 002) 1:2000. After an overnight incubation period at 4 °C, membranes were washed with TBST and treated with secondary antibodies conjucted with alkaline phosphatase (Agrisera, SE) for 1.5 h (dilution 1:2000). Specific proteins were visualized by soaking membranes in 20% BCIP/NBT (Sigma-Aldrich, USA) solution for 1 min. After drying, membranes were scanned with an office scanner. Analyses were performed in triplicate. Densitometric analyses were performed with Image J (NIH, US).

### Chlorophyll concentration determination

Chlorophyll concentration was measured according to Barnes et al. (1992).Chlorophyll *a* and *b* absorbance was measured at 665 and 648 nm with a spectrophotometer (BioTek, Q 5000, US).

### Statistical analyses

Statistical significance was determined with Student’s *t* test (*p* ≤ 0.05), with the exception of *V. lychnitis* survivability results, for which data were arranged in a contingency table and the chi-square test was used. Additionally, a two-way ANOVA was performed to evaluate the effect of double inoculation on plant biomass. Data normality and variance homogeneity were evaluated by the Shapiro-Wilk and Levine’s tests, respectively. Statistical analyses were conducted using STATISTICA ver. 10 (Statsoft).

## Results

### Biometric measurements

**Fig. 1:**
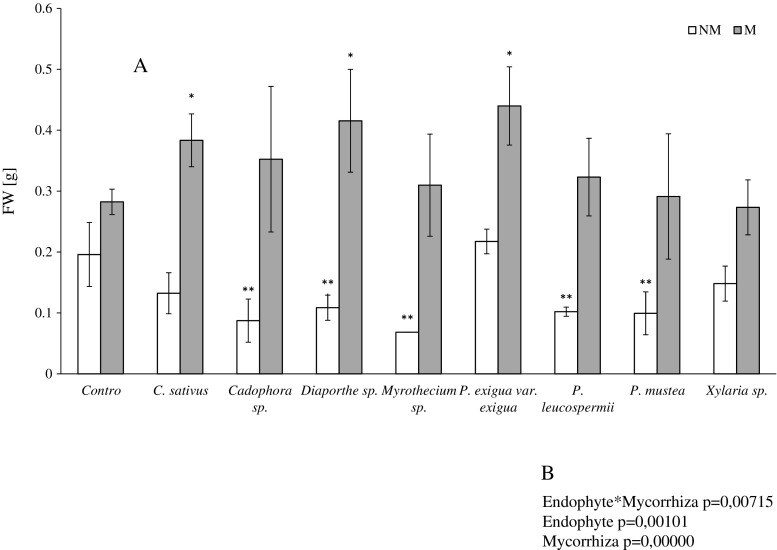
Fresh weight (FW) of *V. lychnitis* inoculated with a single endophyte (*white bars*; NM) and subject to inoculation with AMF and endophytic fungi (*gray bars*; M). *Single stars* indicate statistically significant differences between *V. lychnitis* inoculated with both types of fungi and the M control. *Double stars* indicate statistically significant differences between the NM control and plants inoculated with a single fungal endophyte. Biomass production was lower in NM than M plants in all of the setups tested. Statistical significance was assessed by the *t* test, *P* ≤ 0.05. Whiskers represent SD from *N* = 3–22 (**a**). Two-way ANOVA was performed to evaluate the effect of double inoculation on plant biomass (**b**)

Inoculation of *V. lychnitis* with *Cadophora* sp., *Diaporthe* sp., *Myrothecium* sp., *P. leucospermii*, and *P. mustea* resulted in a significant decrease in biomass production. *Xylaria* sp., *C. sativus*, and *P. exigua* var. *exigua* did not affect *V. lychnitis* growth (Fig. 1a). The addition of mycorrhizal fungi to the setup significantly affected all of the plant-endophyte consortia: M plants yielded more biomass compared to their solely endophyte-inoculated counterparts. The effect of mycorrhiza also was beneficial for control non-inoculated plants. Dual inoculation with AMF and *C. sativus*, *Diaporthe* sp., and *P. exigua* var. *exigua* gave the greatest biomass yield (statistically significant increase compared to the M control), whereas inoculation with the remaining endophytes did not significantly affect the growth of *V. lychnitis* compared to the M control.

**Fig. 2:**
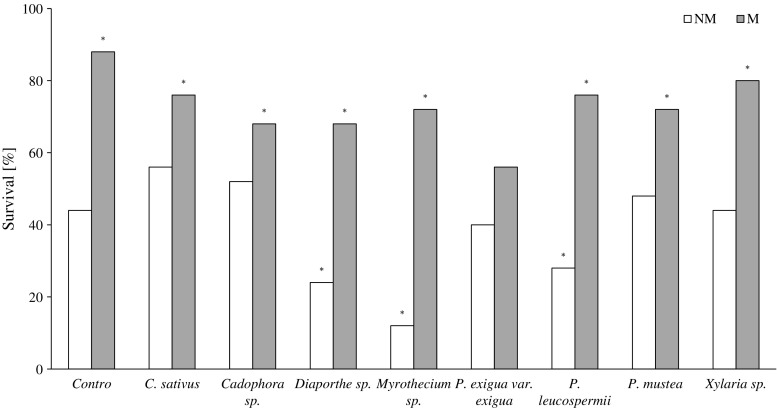
The survival percentage of *V. lychnitis* inoculated with an endophytic fungus (*white bars*; NM) and co-inoculated with AMF and an endophytic fungus (*gray bars*; M) through 24 weeks of growth in substratum from the mine dump “Bolesław.” *Stars* indicate statistically significant differences between inoculated *V. lychnitis* and the NM control. The results were arranged in a contingency table and the chi-square test was used to evaluate statistical significance, *P* ≤ 0.05

Survivability of NM plants inoculated with *Diaporthe* sp., *Myrothecium* sp., and *P. leucospermii* was significantly decreased. *Cadophora* sp., *C. sativus*, *P. exigua* var. *exigua*, *Xylaria* sp., and *P. mustea* did not affect the survival of NM plants. Co-inoculation with AMF significantly improved the survival rate of *V. lychnitis*. In all of the plant-fungi constoria tested except *P. exigua* var. *exigua*, the number of dual-inoculated plants that survived the experiment was significantly higher compared to the NM control (Fig. 2).

### Selection of plant-fungi consortia for further analysis

For more detailed analysis of the interaction between the plant with fungal endophytes and the plant-endophyte-AMF association, plant-microbe consortia were selected according to the biomass production and survivability results. *Xylaria* sp. was selected because it was the only species that did not affect the plants’ biomass production in both single and co-inoculation experiments, nor did it alone influence the survivability of *V. lychnitis*. Single inoculation with *P. exigua* var. *exigua* neither affected plant growth nor survivability, but co-inoculation with AMF resulted in the highest plant biomass yield among the endophytic fungi tested. It also was the only species that did not improve plant survivability in co-inoculation experiments. The responses of *V. lychnitis* to *Diaporthe* sp. were the most diverse among the tested endophytes, depending on the presence of AMF. In single inoculation experiments, *Diaporthe* sp. clearly hindered plant growth and survivability, but co-inoculation with AMF significantly improved all biometric parameters tested. The selection of *Xylaria* sp., *P. exigua* var. *exigua* and *Diaporthe* sp. allowed us to examine the response of the photosynthesis apparatus to fungi that affect plant productivity differently, depending on co-inoculation. We anticipated that alterations of growth would reflect photosynthesis efficiency and pigment/structural protein composition of photosystem II.

### AM fungi and endophyte colonization

**Fig. 3:**
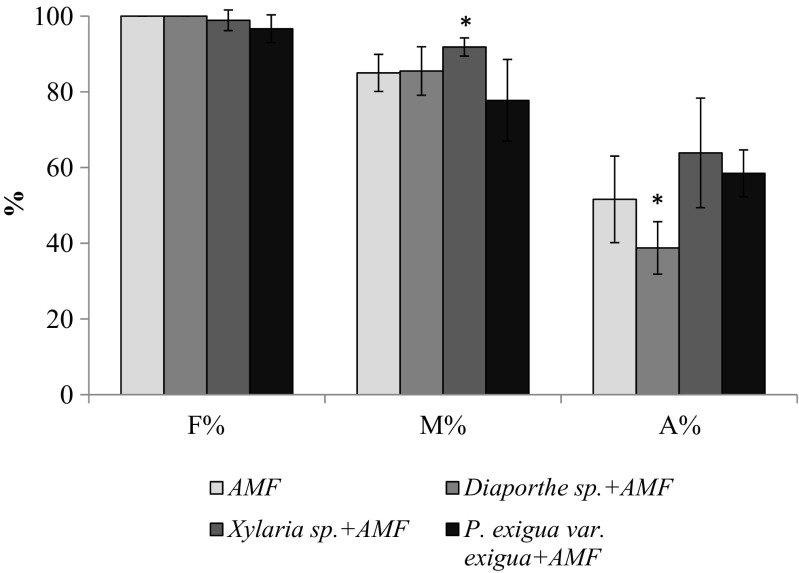
Effect of endophytes (*Diaporthe* sp., *Xylaria* sp. and *P. exigua* var. *exigua*) on arbuscular mycorrhizal colonization parameters of *V. lychnitis* inoculated with *R. irregularis* (AMF). The depicted parameters arethe following: *F (%)* mycorrhizal colonization frequency, *M (%)* relative mycorrhizal colonization intensity, and *A (%)* arbuscule abundance. Plants were cultivated in a greenhouse in substratum composed of a mixture of tailings material, sand, and expanded clay at 3:1:1(*v*/*v*/*v*). The numbers are given in percent according to the parameter definitions by Trouvelot et al. (1986). *Bars* represent mean values from 6 independent measurements, *N* = 6. *Asterisks above the columns* indicate statistically significant differences (*t* test) between each treatment and M control (*P* ≤ 0.05)

Among *V. lychnitis*: (1) non-inoculated; (2) inoculated with endophytes *Diaporthe* sp., *Xylaria* sp., or *P. exigua* var. *exigua*; (3) inoculated with *R. irregularis* and endophytes *Diaporthe* sp., *Xylaria* sp,*.* or *P. exigua* var. *exigua*; and (4) inoculated only with *R. irregularis*, arbuscular mycorrhizas with arbuscules and abundant vesicles were observed in all mycorrhiza-inoculated plants. In plants inoculated with fungal endophytes, thin hyphae of *Diaporthe* sp., *Xylaria* sp., and *P. exigua* var. *exigua* were observed in roots (Fig. S1) growing narrowly between cortical cells. Their dyeability, however, was lower than that of AMF hyphae from the same area of the root. Mycorrhizal colonization parameters are presented in Fig. 3. Mycorrhizal colonization (M%) of *V. lychnitis* inoculated with *Xylaria* sp*.* exhibited a significant increase (7%) in comparison to AMF control plants. M plants inoculated with *Diaporthe* sp. showed a significant decline (13%) in A%. In root samples of NM *V. lychnitis*, no mycorrhizal structures and endophytes were found.

### Photosynthesis efficiency

**Fig. 4:**
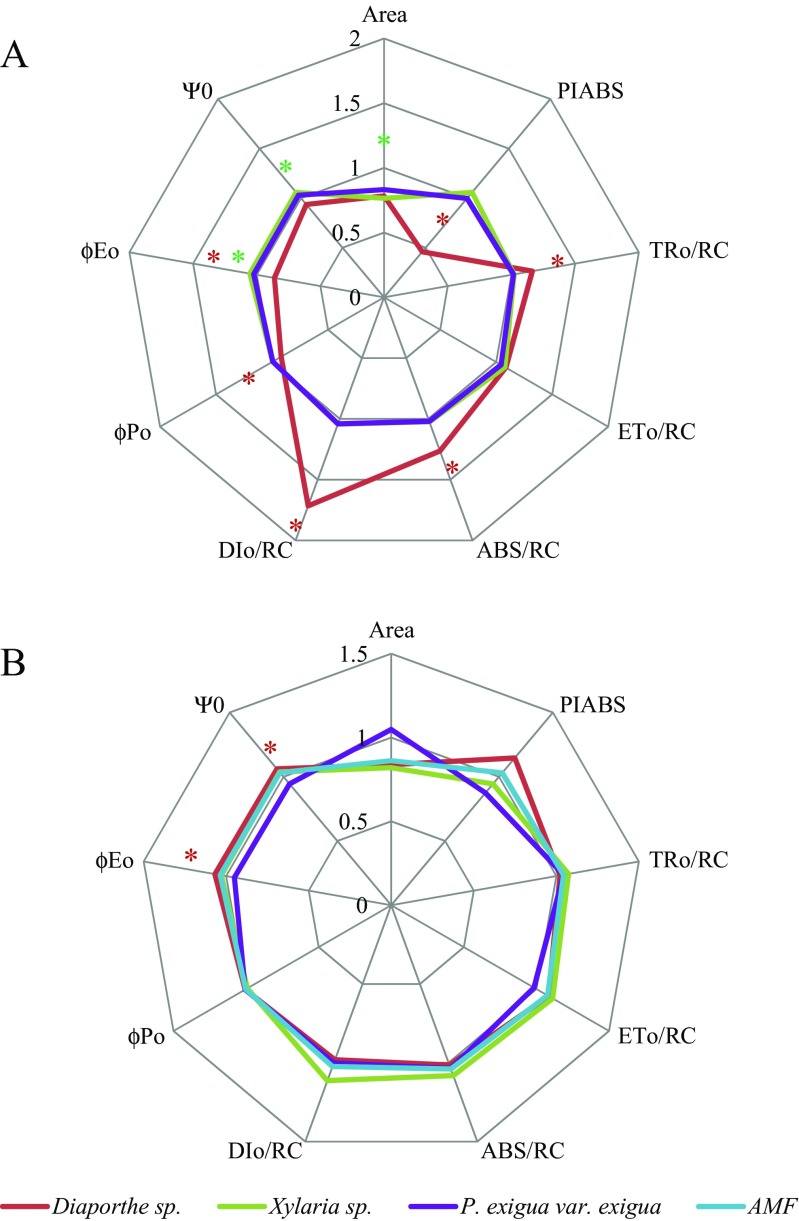
“Spider plots”of selected fluorescence parameters describing energy fluxes in PSII in **a** plants inoculated with endophytes and **b** plants inoculated with endophytes and *R. irregularis* (AMF) The values were normalized to those of the NM control plant (i.e., NM controls have a value of 1 for each parameter). Definitions of the parameters in Table 1 *Asterisks* in colors corresponding to the line for each fungus indicate statistically significant (*t* test) differences between each treatment and the NM control (*P* ≤ 0.05). *Whiskers* represent SD from *N* = 5

Among the endophytes selected for further analysis, single inoculation with *Diaporthe* sp. resulted in significant declines (compared to the NM control) in PI_ABS_ (54%), ϕPo (7%), and ϕEo (11%) and an upregulation of TRo/RC (12%), ABS/RC (20%), and Dio/RC (51%). *Xylaria* sp. inoculated plants showed a significant reduction of the area parameter (19%) and a significant increase of Ψ0 (8%), ϕEo (9%), and a 21% in PI_ABS_ in comparison with NM control plants. Photosynthesis efficiency was not affected significantly by inoculation with *P. exigua* var. *exigua* (Fig. 4a).

In M plants, inoculation with *Diaporthe* sp. induced a significant increase (compared to NM control plants) of ϕEo and eΨo (both 7%) which translated into an upregulation of PI_ABS_. Co-inoculation with *Xylaria* sp. or *P. exiqua* var. *exigua* and AMF did not affect photosynthesis efficiency (Fig. 4b).

### Rubisco and photosystem II protein abundance

**Fig. 5:**
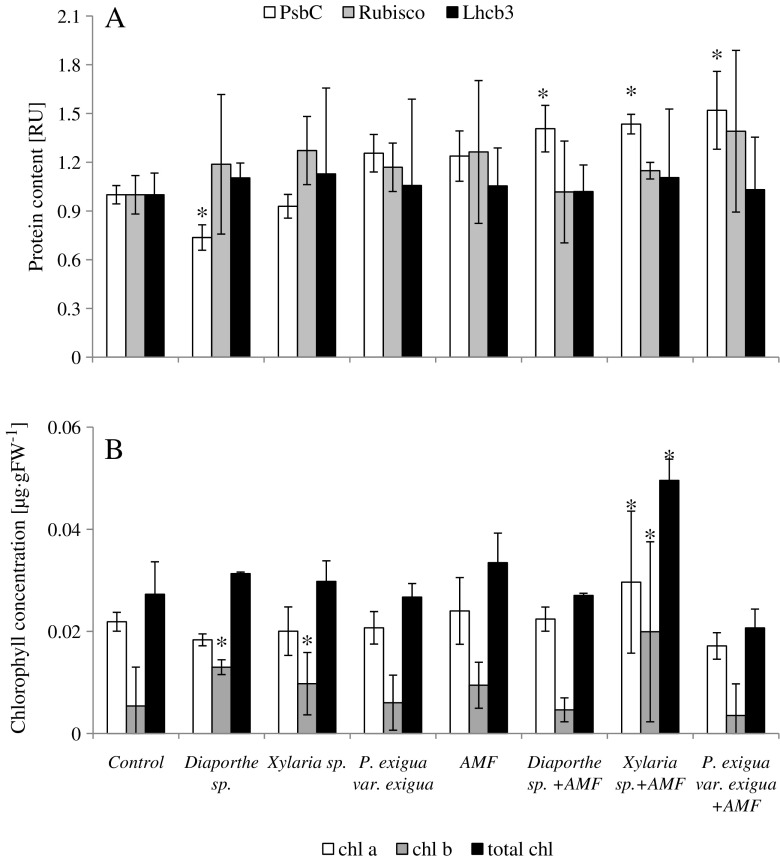
**a** Relative PsbC, Rubisco, Lhcb3 content from *V. lychnitis*. *Bars* represent mean values from three independent measurements, *N* = 3. The values were normalized relative to those of the NM control plant. **b** Total chlorophyll, chlorophyll a and b concentrations from *V. lychnitis*. *Bars* represent mean values from seven independent measurements, *N* = 7. Plants were inoculated with endophytes and/or inoculated with *R. irregularis* (AMF) *Asterisks above the bars* indicate statistically significant differences (*t* test) between each treatment and the NM control (*P* ≤ 0.05). *Whiskers* represent SD

Among plants inoculated with endophytes, the abundance of PsbC was decreased by 30% in *Diaporthe* sp-colonized plants versus NM controls. *Xylaria* sp. and *P. exigua* var. *exigua* did not significantly affect the abundance of PsbC. Co-inoculation with AMF significantly increased PsbC abundance in all plants tested. *Diaporthe* sp., *Xylaria* sp., and *P. exigua* var. *exigua* increased PsbC abundance by 40, 43, and 52%, respectively, compared to NM control plants. No significant differences in Rubisco and in Lhcb3 abundance were found in colonized plants, compared to NM control plants (Fig. 5a).

### Chlorophyll concentration

In NM plants inoculated with *Diaporthe* sp. and *Xylaria* sp., a significant increase (2.5-fold and 2-fold, respectively) in Chl b concentration, compared to NM control plants, was observed (Fig. 5b)*. P. exigua* var. *exigua* did not affect Chl*b* concentration (Fig. 5b). No differences in total chl and Chl*a* were observed in NM plants. In M plants inoculated with *Xylaria* sp., a significant increase in total Chl (almost 2-fold increase), in Chl a (0.5-fold), and in Chl b (almost 4-fold), compared to NM control plants, was found. In the remaining treatments, no significant differences were observed (Fig. 5b).

## Discussion

The role of fungal endophytes in plant physiology and their possible utilization as growth promotion and stress protection agents recently has been a popular subject of research. Here, we report that, under laboratory conditions, fungal endophytes did not promote *V. lychnitis* growth in substrate from a post-mining waste dump. In single inoculation experiments, plant fitness, in terms of biomass production and leaf area (data not shown), was negatively affected by 5 out of the 8 endophytes tested. The remaining three fungi had no effect on plant growth. It seems that reduced growth was the cost of maintaining the endophytes within plant tissues. AMF in this setup remarkably improved the growth parameters of the endophyte-inoculated plants. In all plant-fungi consortia examined, biomass production and survivability (excluding *P. exigua* var. *exigua*) was improved compared to the NM control, suggesting that AMF positively modulates the plant-fungal endophyte interaction. *V. lychnitis* yielded the highest biomass when co-inoculated with AMF and *C. sativus*, *Diaporthe* sp., and *P. exigua* var. *exigua.* The survival rate of M plants co-inoculated with endophytes was notably higher than the NM control, but not as high as the AMF control (with no endophyte colonization). This probably can be attributed to the methodology used in the experiment. Inoculation with the endophyte took place under in vitro conditions, 4 weeks before inoculation with AMF to ensure endophyte colonization. It seems as though that the attenuating effect of AMF was not sufficient enough to allow all the plants inoculated with endophytes 4 weeks earlier to survive. In order to gain a comprehensive understanding of the endophyte-induced growth dynamics, a different experimental setup (simultaneous inoculation with AMF and fungal endophytes) is necessary. *V. lychnitis* is a biennial herb, which grows up to 2 m in height in its second year. To describe the role of symbiotic fungi in *V. lychnitis* fitness, more thorough analysis of plant growth, seed production, etc. after two growth seasons is required. Another issue that needs to be raised is that *V. lychnitis* in natural conditions is almost exclusively found associated with mycorrhiza (unpublished data). This suggests that any depletion of the mycorrhizal partner may have negatively affected the plant’s physiology, which, in turn, may have affected its interaction with the endophyte. Overall, *V. lychnitis* could only benefit from the endophytic fungi in its mycorrhizal state.

The mechanisms by which mycorrhizas improve plant growth and fitness under metal toxicity have been reviewed recently (Ruytinx et al. 2016). The majority of data describing the beneficial role of AMF under metal toxicity comes from experiments conducted under controlled conditions, or that did not recognize the potential role of other symbiotic organisms in mycorrhiza-dependent toxic metal stress alleviation, even though in natural environments plants are inhabited by a multitude of microorganisms. In this study, we examined the growth response of *V. lychnitis* in both single inoculation experiments and in co-inoculation experiments with both AMF and fungal endophytes. We found that co-inoculation with certain species may facilitate plant growth in a toxic metal-enriched environment. The models used in this study do not exhaust all the possible interactions of *V. lychnitis* in nature, but are a step forward in understanding the role of different microorganisms in plant biology. To elucidate the mechanisms of this tripartite interaction, further research is necessary.

According to the available literature, the fungal endophytes tested in this study usually establish beneficial or neutral associations with their hosts. Being one of the most dominant endophytes, *Diaporthe* (*Phomopsis*) species are commonly isolated as endophytes from different dicot plant hosts (Suryanarayanan et al. 2002; Sun et al. 2011). Some species of *Diaporthe*, however, show either pathogenic or mutualistic interactions with plants depending on the host and its health; for example, *D. phaseolorum* is pathogenic to soybean (Santos et al. 2011), but endophytic in mangroves (Sebastianes et al. 2011). *Diaporthe* spp. have been reported to cause a wide range of plant diseases (Scheper et al. 2000; Heller and Gierth 2001). *Xylaria* species occur on a broad diversity of plant hosts; however, they are frequently isolated from wood tissues (Frohlich et al. 2000; Wang et al. 2014). Fungi from this genus present fungal activity against plant pathogens (Park et al. 2005; Fukasawa et al. 2009) but also show strong capacity to degrade cellulose and lignin (Osono and Takeda 2002). This strategy may be adapted for a saprotrophic lifestyle. Species from the *Phoma* genus are asymptomatically present in numerous plants: Junker et al. (2012) isolated this fungus along with other fungi from wild *Arabidopsis thaliana* in which it did not cause any disease symptoms. In a single inoculation in vitro experiment, however, *Phoma* sp. parasitized *Arabidopsis*, suggesting that the *A. thaliana*-*Phoma* sp. interaction is modulated by the plant microbiota (in vitro conditions may had also affected the interaction). A similar relationship was reported by Larimer et al. (2010) who showed that fungal foliar endophytes decreased plant performance; however, co-inoculation with AMF attenuated the negative effect of endophytes.

The growth promoting role of fungal endophytes, under limited resource availability, is controversial. Studies performed on *Festuca* grass species and the *Neothyphodium/Epichloe* endophytes give conflicting results (Ahlholm et al. 2002). In Ahlholm et al.’s (2002) experiment, fungus species previously described as neutral and mutualistic endophytes severely affected the growth of their host. Endophytic fungi acquire all necessary growth and maintenance resources from their host plant, thereby affecting resource transport from source to sinks and allocation within the plant (Ahlholm et al. 2002). Vegetation in conditions of limited resource availability, such as a post-mining waste dump, severely hinder plant performance. An additional sink probably exceeded the host ability to maintain its own vegetative functions. The potential benefits coming from the presence of the endophyte did not compensate the costs of the symbiosis leading to decreased plant biomass production. In contrast to fungal endophytes, AMF hyphae extend beyond the rhizosphere. This allows AMF to aquire resources independent of plant growth. Mineral nutrients acquired by the fungi may be translocated to the plant. We speculate that the endophyte confined to plant tissues was not efficient in provisioning its host, which resulted in growth retardation. The presence of the AMF in the setup remarkably improved plant growth, suggesting that the benefits (nutrient acquisition, stress protection) provided by the AMF were sufficient to allow the plant to provide nutrients to all its symbiotic partners. Additionally, a role in modulating endophytic fungi growth and lifestyle by AMF cannot be excluded, but this requires further research.

Toxic metals significantly affect the performance of the photosynthetic apparatus, limiting the efficiency of carbon assimilation, thus affecting the overall nutritional status of the plant. Limiting the production of the “source” forces the “sinks” to adapt. An additional energy sink, the endophyte, requires the plant to share its carbon resources with its symbiotic partner. Under optimal conditions, plant production may exceed its needs, allowing it to allocate a share of its photoassimilates to the fungi. In photosynthesis limiting conditions, however, the plant suffers from carbon deficiencies (with a consequent slower growth rate, diminished ability to counteract stress-induced damage, etc.) hindering its ability to invest in additional sinks for resources. Aloui et al. (2011) showed that *Glomus irregulare* alleviated the negative effects of Cd on photosynthesis in *Medicago truncatula* by increasing the plant’s ability to utilize light energy and by facilitating electron transport. According to Rozpądek et al. (2015), colonization of orchard grass by *Epichloe typhina* led to changes in the host’s photosynthetic apparatus, e.g., increased electron density flux, increased stomatal conductance, and an abundance of Chl*b* and PSI and PSII proteins. As a result, PSII photochemistry efficiency, carbon assimilation, and light harvesting capacity in plants were improved, allowing orchard grass to cope with endophyte energy demands while also sustaining orchard grass growth.

In single inoculation experiments with *Diaporthe* sp., *V. lychnitis* exhibited a significant decline in photosynthesis performance. The efficiency of energy production in relation to absorption, manifested as electron flow in PSII (PI_ABS_), decreased by close to 50%. The photosynthesis index PI_ABS_ combines three variables indicative of different processes associated with PSII photochemistry: the density of reaction centers, the quantum yield of primary photochemistry, and the ability to transfer electrons from PSII to PSI (Corrêa et al. 2006). In single inoculation with *Diaporthe* sp., the photochemistry of PSII was decreased due to a significant decline in the efficiency of primary photochemistry (φEo, φPo). Even though, the light-harvesting capacity was improved (ABS/RC), the photosynthetic apparatus was unable to transform incident light into energy (lower electron transport efficiencies) necessary for carbon assimilation. These results suggest that even though solar energy was absorbed, it was not transformed into fixed energy, but instead was dissipated as heat (DIo/RC). Interestingly, inoculation with mycorrhiza (co-inoculation with AMF) restored to control levels, or even above control levels, all the photosynthesis indicies that were negatively affected by single inoculation with *Diaporthe* sp. The restoration was correlated with a significant growth improvement. In the case of *Xylaria* sp.-inoculated plants (single inoculation) which did not respond to the endophyte growth-wise, a significant improvement in φEo and Ψ0 was found, but this did not translate into elevated overall photosynthesis efficiency (PI_ABS_). According to previous reports, inoculation with mycorrhiza improves plant growth in toxic metal-enriched habitats. Higher biomass yield often is correlated with improved photosynthesis performance (Rozpądek et al. 2014, reviewed in Ruytinx et al. 2016). In this study, however, mycorrhizas did not affect the photosynthesis efficiency of *V. lychnitis*, suggesting that accelerating the carbon assimilation rate was not necessary for improved growth. It also was not required for provisioning its symbiotic partners, indicating that the energy demands of the plant-microbe consortium were met by means other than improved photosynthesis efficiency.

The role of toxic metals in chlorophyll (Chl) biosynthesis and stability has been comprehensively described. Toxic metals were reported to inhibit chlorophyll biosynthesis (Ouzounidou 1995). Pb was reported to inhibit chlorophyll biosynthesis by causing impaired uptake of essential elements such as Mg and Fe by plants (Burzyński and Grabowski 1984). Chl*b* is more vulnerable to Pb toxicity than Chl*a* (Vodnik et al. 1999). Pb changes the lipid composition of thylakoid membranes (Stefanov et al. 1995) and damages the photosynthetic apparatus due to its affinity for protein N- and S-ligands (Ahmed and Tajmir-Riahi 1993). Cd is known to decrease the total Chl*a/b* ratio in plants (Sheoran et al. 1990) and to suppress chloroplast development (Malik et al. 1992a, b). Here, inoculation with endophytes *Xylaria* sp. and *Diaporthe* sp. significantly increased the abundance of Chl*b*. *Diaporthe* sp. also increased energy absorption and flow through a single reaction center (although it did not translate into energy production). Chl*b* plays an important role in the assembly of light-harvesting complexes and in the structure of reaction centers. In a previous study, fungal endophytes were found to alter the composition of photosynthetic pigments and light harvesting center proteins (LHC), including the relative abundance of Chl*b*, PsbC, and other proteins involved in light harvesting and processing. This was accompanied by improved photosynthesis efficiency (Rozpądek et al. 2015). Higher energy fluxes potentially accelerate energy production in PSII. In this experiment, however, probably due to plant growth conditions, structural changes did not translate into enhanced performance. In order to map endophyte-induced changes in the composition of photosynthesis functional proteins, we tested those shown previously to be affected by *Dactylis glomerata* inhabited by *E. typhina* and others. Unfortunately, due to methodology issues (the antibodies available did not react with epitopes from *V. lychnitis* proteins or did not allow us to perform a semi-quantitative analysis), we were not able to quantify the abundance of other photosynthesis-related proteins. Those that we quantified did not exhibit significant changes upon inoculation. Our results indicated that only the abundance of the LHC structural PsbC protein increased in M plants co-inoculated with endophytes, but not in single inoculation experiments with endophytic fungi or AMF. Because inoculation with the AMF alone did not yield such results, we propose that increasing the abundance of PsbC is an endophyte-specific feature, but in the case of *V. lychnitis*, only plants co-inoculated with AMF (*V. lychnitis* is mycorrhizal in nature) exhibit such an alteration. The abundance of the remaining proteins (Rubisco LSU and Lhcb3) evaluated in this study did not change in any of the experimental setups. This suggest that even though the presence of the endophytes did affect the composition of photosynthetically active pigments and proteins in *V. lychnitis*, these changes were insufficient to improve photosynthesis performance.

## Conclusions

Studies investigating interactions between fungal endophytes, AMF, and non-grass hosts have received little attention until recently. The experiments conducted here showed that *V. lychnitis* did not benefit from single inoculation with endophytes in a substrate containing toxic metals. Inoculation with both AMF and endophytes significantly improved plant growth parameters. Particularly, *V. lychnitis* co-inoculated with AMF and *C*. *sativus*, *Diaporthe* sp., and *P. exigua* var. *exigua* yielded the highest biomass, exceeding the growth rate of both non-inoculated and AMF plants. These results, hopefully, can be utilized in phytoremediation strategies. Inoculation with the fungi tested did not affect photosynthesis efficiency. Interestingly, however, in the beneficial setups that included fungal endophytes, the abundance of PsbC was increased. This indicates that upon colonization with endophytic fungi, changes in the composition of proteins involved in light harvesting do take place. Previously, this has been found to be associated with an improvement of photosynthesis efficiency.

## Electronic supplementary material

Supplement Fig. S1 (A) *Xylaria* sp. (arrow) growing between root cells of mycorrhizal *V. lychnitis.* (B) *P. exigua* var. *exigua* within root cells (arrow) of mycorrhizal *V. lychnitis*. (C) Mycorrhizal *V. lychnitis* roots inoculated with *Diaporthe* sp. (arrows) showing hyphae of endophytic fungi between root cells. Roots were stained with aniline blue (DOCX 497 kb)

Supplement Fig. S2 (A) Plants inoculated with endophyte *P. leucospermi*. (B) Plants cultivated in pots. (DOCX 366 kb)
