# Supplementary figures and images for: Interactions of arbuscular mycorrhizal and endophytic fungi improve seedling survival and growth in post-mining waste
Source: Mycorrhiza. 2017 Mar 20;27(5):499–511. doi: 10.1007/s00572-017-0768-x (PMC5486607; doi:10.1007/s00572-017-0768-x)

Fig. S1


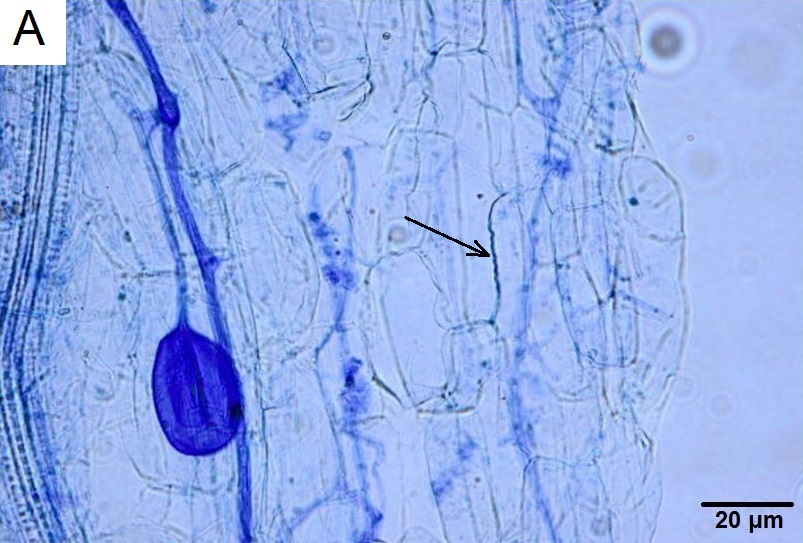


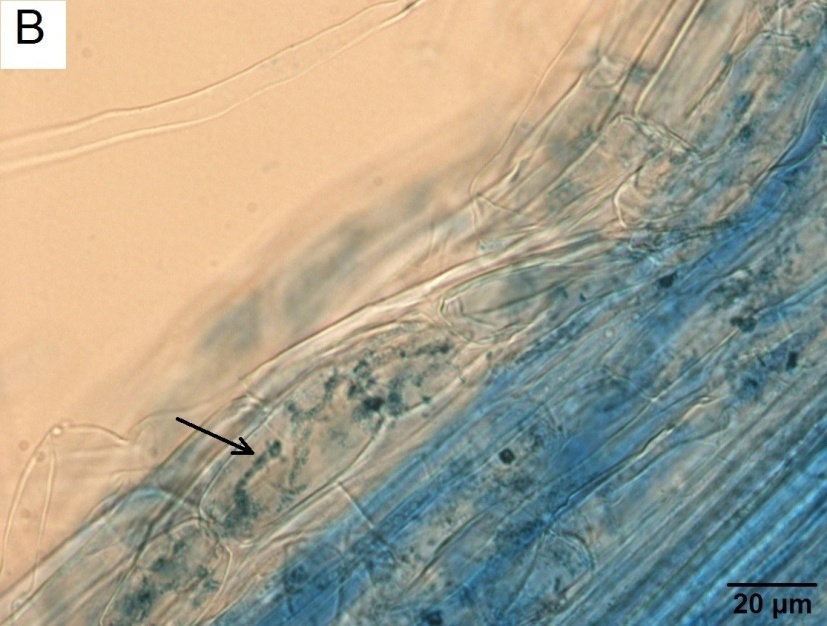


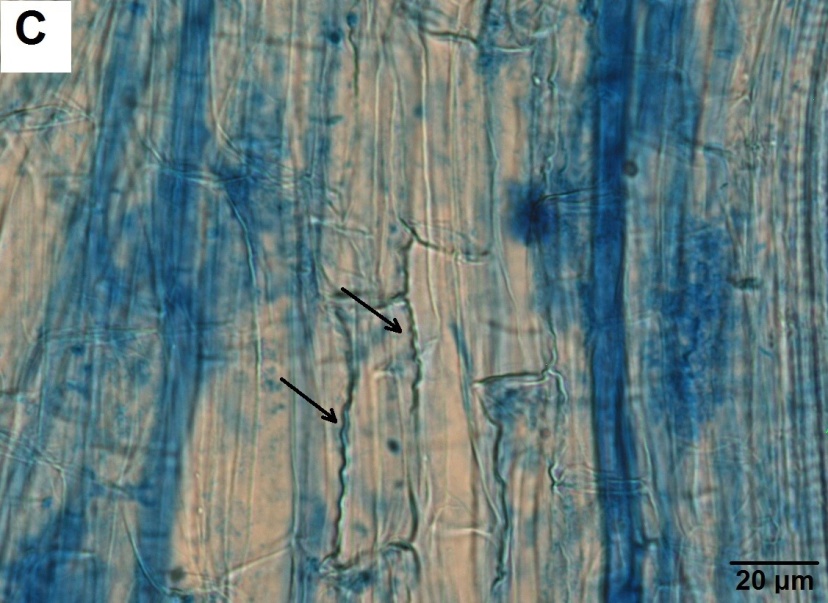

Supplement: Supplementary file 1 — (A) Xylaria sp. (arrow) growing between root cells of mycorrhizal V. lychnitis. (B) P. exigua var. exigua within root cells (arrow) of mycorrhizal V. lychnitis. (C) Mycorrhizal V. lychnitis roots inoculated with Diaporthe sp. (arrows) showing hyphae of endophytic fungi between root cells. Roots were stained with aniline blue (DOCX 497 kb) [file 572_2017_768_MOESM1_ESM.docx]

Fig. S2


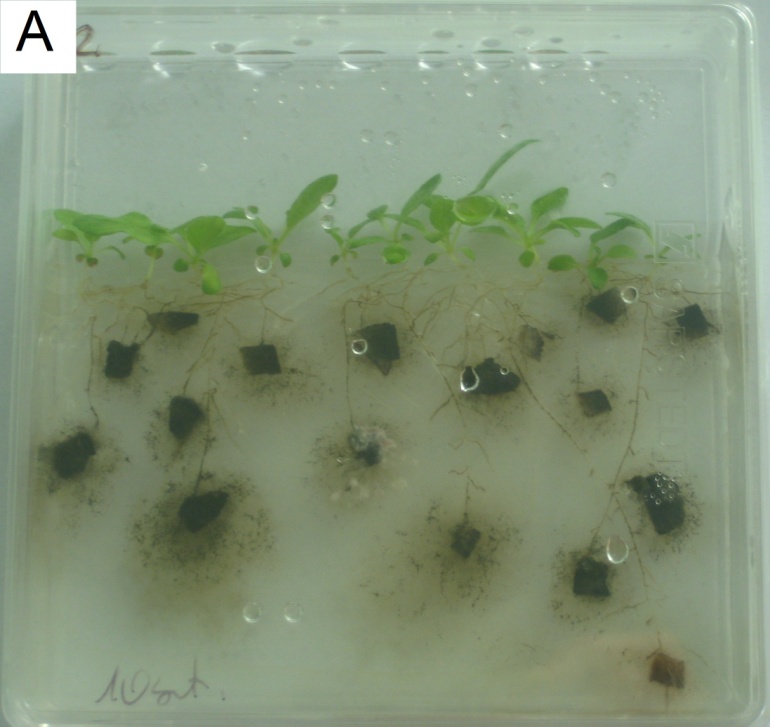


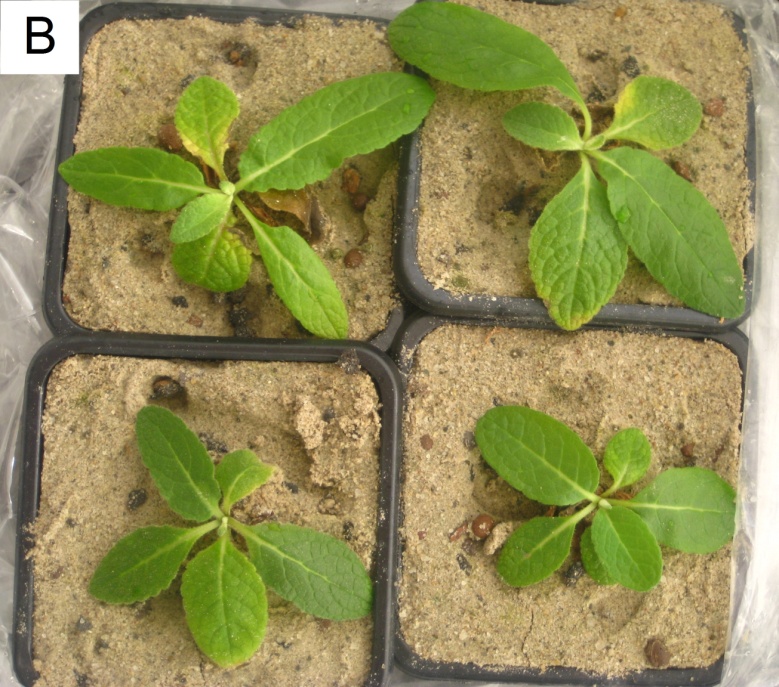

Supplement: Supplementary file 2 — (A) Plants inoculated with endophyte P. leucospermi. (B) Plants cultivated in pots. (DOCX 366 kb) [file 572_2017_768_MOESM2_ESM.docx]
